# Relevance of tumor boards for the inclusion of patients in oncological clinical trials

**DOI:** 10.1007/s00432-022-04559-0

**Published:** 2023-03-30

**Authors:** Hendrik Dapper, Maurice Dantes, Peter Herschbach, Hana Algül, Volker Heinemann

**Affiliations:** 1 Comprehensive Cancer Center Munich, Klinikum rechts der Isar, Technical University Munich, Munich, Germany; 2 Department of Radiation Oncology, Comprehensive Cancer Center Munich, University Hospital Munich, LMU Munich, Munich, Germany; 3 Comprehensive Cancer Center München, Institute for Tumor Metabolism, Klinikum rechts der Isar, Technical University of Munich, Munich, Bavaria Germany; 4 Department of Medicine III, Comprehensive Cancer Center Munich, University Hospital Munich, LMU Munich, Munich, Germany

**Keywords:** Tumor boards, Oncological clinical trials, Patient recruitment

## Abstract

**Introduction:**

Major national and international oncological societies generally recommend treating a significant proportion of oncological patients in clinical trials to improve therapy strategies for cancer patients. At cancer centers, the recommendation about the appropriate therapy for the individual tumor patient is usually made in interdisciplinary case discussions in multidisciplinary tumor boards (MDT). In this study, we examined the impact of MDTs for the inclusion of patients in therapy trials.

**Methodology:**

A prospective, explorative study of the Comprehensive Cancer Center Munich (CCCM) was conducted at both university hospitals in 2019. In the first phase, various MDTs’ case discussions about oncological situations and their decisions regarding possible therapy trials were recorded in a structured manner. In the second phase, the actual inclusion rates of patients in therapy trials and reasons for non-inclusion were examined. Finally, the data of the respective university hospitals were anonymized, pooled and analyzed.

**Results:**

A total of 1797 case discussions were reviewed. Therapy recommendations were made in 1527 case presentations. 38 (2.5%) of 1527 patients were already included in a therapy trial at the time of case presentation. The MDTs recommended inclusion of an additional 107 cases (7%), for a therapy trial. Of these patients, 41 were finally enrolled in a therapy trial which resulted in a total recruitment rate of 5.2%. Despite MDTs’ recommendations, 66 patients were not included in a therapy trial. The main reason for non-inclusion was insufficient inclusion or existing exclusion criteria (*n* = 18, 28%). In 48% of all cases (*n* = 31), the reason for non-inclusion could not be determined.

**Conclusion:**

The potential of MDTs as an instrument for the inclusion of patients in therapy trials is high. To increase the enrollment of patients in oncological therapy trials, structural measures such as the central use of trial administration and MTB software in addition to standardized tumor board discussions must be established to ensure a seamless flow of information about actual recruiting trials and the current status of trial participation of patients.

## Introduction

Oncological centers of excellence are characterized by three central attributes: multidisciplinary patient treatment with therapy definition primarily in discussions in multidisciplinary tumor boards (MDTs), the treatment of patients in oncological therapy trials, including translational research projects, and networking with local oncological care providers (Prognos 2022).

Therapy studies review the promising developments in preclinical and clinical research in light of the benefits for the relevant patient population (Krzyzanowska et al. 2011). New standards emerge from trial results and form the basis in oncological decision-making processes. The implementation of large, randomized oncological therapy trials, therefore, has both an epidemiological relevance regarding the creation of new therapy standards for entire patient collectives, as well as an individual significance for the patient treated within the framework of such a trial (Schwentner et al. 2012; Zaharoff and Cipra 2018). Major national and international oncological societies such as the German Cancer Aid (Deutsche Krebshilfe, DKH) or the European Organization for Research and Treatment of Cancer (EORTC), generally recommend treating a significant proportion of oncological patients in clinical trials (Casali et al. 2015; Nass and Io 2010). For example, for the funding priority program "Oncological Centers of Excellence", the DKH requires treatment within the framework of clinical trials for 90% of pediatric carcinoma patients, for 50% of patients with hematological and lymphatic tumors and for 10% of patients with solid tumors (Deutsche Krebshilfe 2022). However, a distinctive drawback of therapy trials as well as the treatment in those trials is the high expenditure of personnel and organizational resources. Therefore, it is generally difficult to treat a significant number of patients in oncological therapy trials.

The decision-making process in determining the therapy of an oncological case is usually carried out within the framework of MDTs based on quality assurance assessments (Saghir et al. 2014; Homayounfar et al. 2022). It has been shown that the exclusive presentation of a case at a MDT can lead to a change in the treatment plan (Charara et al. 2017). Retrospective investigations also indicate that the oncological outcome for patients can be positively influenced solely due to an interdisciplinary case discussion that has taken place at a MDT (Saghir et al. 2014; Charara et al. 2017; Deutsche Krebsgesellschaft 2022). Therefore, Oncology centers assume case discussions at MDTs are routine. The respective therapy recommendations made with interdisciplinary consensus are generally widely accepted by specialists and patients due to the resulting high degree of quality assurance (Petty and Vetto 2002).

Case presentations at MDTs can significantly lead to a higher trial participation rate (Mobley et al. 2020; Kuroki et al. 2010). The high rate of case presentations at MDTs at oncology centers reflects influences on the recruitment of patients into therapy trials. In Germany, the management of individual therapy trials is predominantly organized decentrally in individual clinics within a center (Federal Ministry of Education and Research 2022). Thus, an exchange of information with the MDTs about the respective trial program is not necessarily given. For oncological care in the context of therapy trials at oncology centers, it is of great interest to which extent the documentation of existing trials is offered and the inclusion of patients into clinical trials takes place via MDTs.

The Comprehensive Cancer Center Munich, as the largest center of excellence in oncology in its region, sets the goal of investigating the documentation structure of the MDTs in relation to therapy trials at the two university hospitals in Munich. At various MDTs, oncological situations and decisions regarding possible therapy trials were to be recorded in a structured manner to generate approaches for optimizing the existing structures and to increase the recruitment rate of tumor patients in therapy trials via the MDTs based on the knowledge gained.

## Methodology

The present study is a prospective, explorative study of the Comprehensive Cancer Center Munich (CCCM), consisting of the CCC of the Ludwig-Maximilians University Hospital Munich (CCCLMU) and the CCC of the Klinikum rechts der Isar of the Technical University of Munich (CCCTUM). The survey was conducted at both university hospitals in 2019.

In an initial three-month observation phase (phase 1), six MDT sessions each of eight MDTs (Thoracic Oncology, Gastrointestinal Oncology, Neurologic Oncology, Gynecologic Oncology & Breast Cancer, Genitourinary Oncology, Head & Neck Oncology, Sarcoma, Hematology Oncology) were evaluated independently at both university hospitals. A standardized evaluation form was completed at each tumor session by a qualified CCCM medical staff member. The following data were recorded:

- Oncological situation and therapy decision:
  o Primary therapy
  p (Neo-)adjuvant/additive
  q Locally treatable recurrence/progression/residual tumor
  r Palliative
  s Insufficient information—no decision on therapy
- Documentation in the MDT with regard to therapy trials:
  o Patient currently already enrolled in therapy trial
  p Inclusion in therapy trial or screening recommended
  q Deviation with regard to inclusion or exclusion criteria
  r No trial available

Therapeutic trials were defined as all pharmacological as well as non-pharmacological, officially registered phase I–IV studies.

If a patient was presented to different boards or more than once during the observation period, only the presentation with a final therapy recommendation was taken into account.

In the second study phase (phase 2), additional follow-up was done on those patients for whom screening for inclusion in a therapy trial was recommended at the MDT. The first step was to find out whether a trial inclusion actually took place. In the event of non-participation in a trial, the reasons why a possible inclusion in a therapy trial ultimately did not occur were determined. The corresponding information was requested from the respective study outpatient departments of the responsible hospitals three months after the respective MDT decision. Reasons for non-inclusion were categorized as follows:

- Inclusion criteria not met/exclusion criteria present
- Patient rejection
- Treatment outside the centers
- Other reasons

**Fig. 1:**
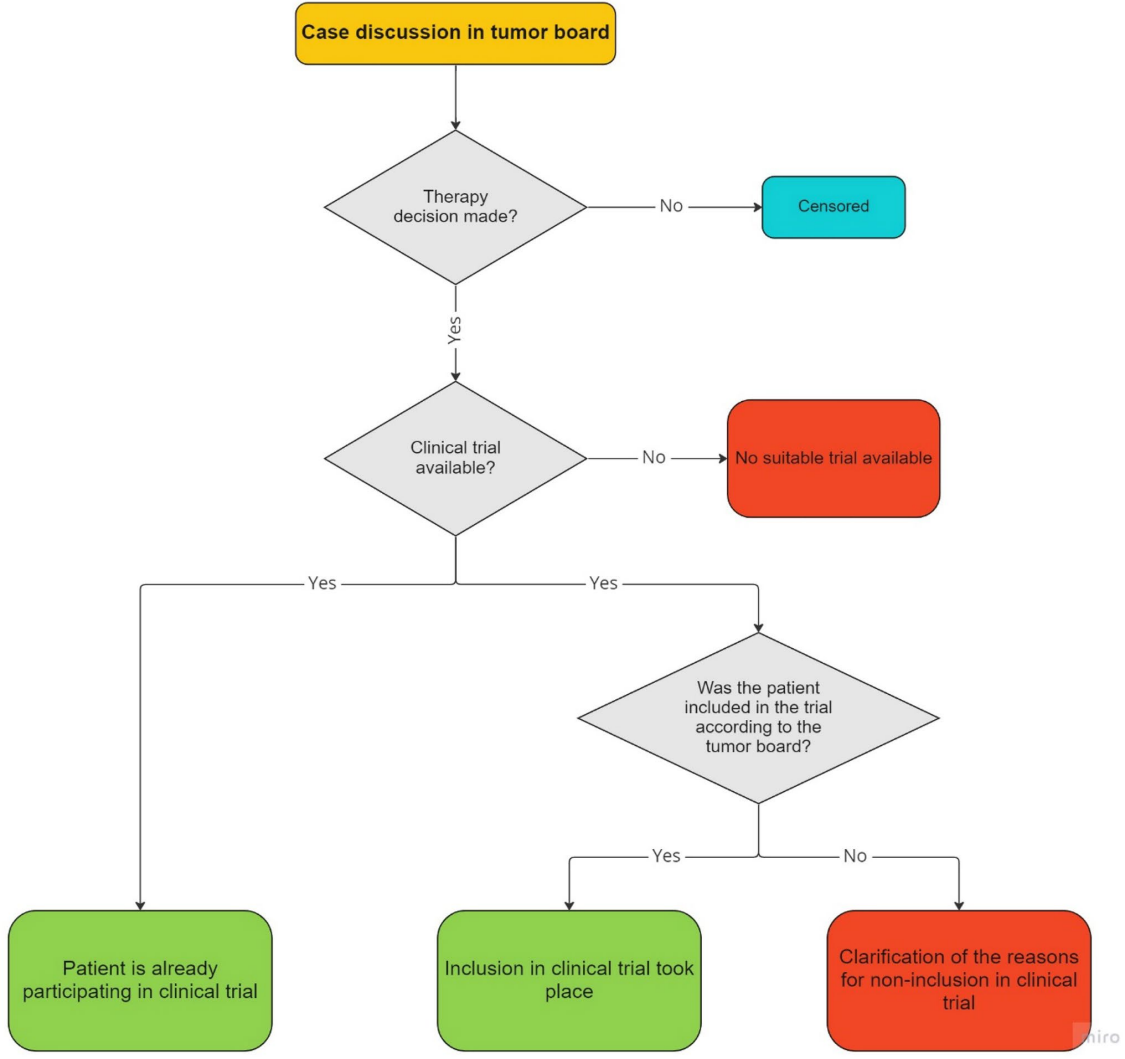
Procedure of the RAISE trial. Documentation and follow-up algorithm of each patient case presented at the tumor board

Figure 1 shows the examination algorithm for each patient case presented at the MDT.

Finally, the data of the respective university hospitals were anonymized, pooled and analyzed centrally by oncological and scientifically active physicians of the CCCM.

The ethics committees of both participating centers approved this research before the start of the collaborative study.

Figures were created with Microsoft Office 2016.

## Results

**Fig. 2:**
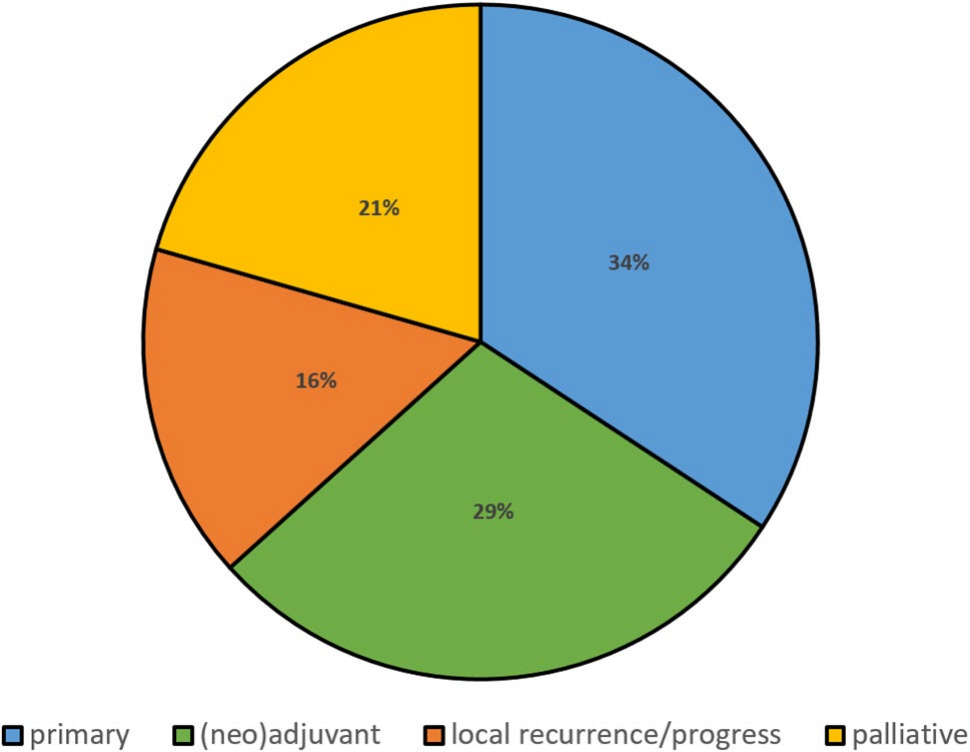
Frequency distribution of oncological cases according to the therapy decisions in the tumor boards

At both CCC-Munich sites, a total of 1797 case discussions were observed in the first study phase. These were investigated over six sessions, each of eight different MDTs (Thoracic Oncology, Gastrointestinal Oncology, Neurologic Oncology, Gynecologic Oncology & Breast Cancer, Genitourinary Oncology, Head & Neck Oncology, Sarcoma, Hematology Oncology). In 270 patients, the tumor board information was insufficient to make an immediate therapy decision (15%). The most frequent causes were incomplete staging and incomplete histology. The indication for the remaining 1527 case presentations was distributed approximately one third each to oncological primary therapy (*n* = 523, 34%) or neoadjuvant/adjuvant treatment (*n* = 444, 29%). Less frequently, there were indications for local treatment due to local recurrence or limited progression (*n* = 246, 16%) and for palliation (*n* = 314, 21%). Figure 2 shows the frequency distribution according to the treatment constellation.

38 (2.5%) of 1527 patients were already included in a therapy trial at the time of case presentation at the MDT according to the given information. In further 107 cases (7%), the MDTs recommended inclusion or screening for a therapy trial. The inclusion or exclusion criteria of existing therapy trials prevented the participation of a further 32 patients (2.1%) whose oncological status and entity affiliation would have been potentially compatible with a therapy trial.

Of the 107 patients mentioned above with recommendations for trial inclusion, 41 were finally enrolled in a therapy trial (38% of all suggestions). Despite MDT recommendations, 66 patients were not included in a therapy trial. Insufficient inclusion or existing exclusion criteria, which were not initially present in the MDT, were the most frequent cause (*n* = 18, 28%). Nine patients (14%) refused to participate in the recommended trial and eight (12%) were ultimately treated externally. In 48% of all cases (*n* = 31), the reason for non-inclusion could not be determined.

Considering the patients already included in therapy trials and those who agreed to a participation option after the corresponding MDT recommendation, a total of 79 trial participants were documented. In relation to the observed cases for which a therapy recommendation was made, this corresponds to a recruitment rate of 5.2% recorded and tracked at the MDTs. More than 80% of patients (*n* = 65) were in the oncological treatment situations (neo)adjuvant, relapse/progress, or palliative. Only about 18% (*n* = 14) of all study participants were in an oncological situation of primary therapy. An overview of the frequency distributions according to the treatment situations, as well as the associated opportunities for participation in therapy trials, actually and total inclusion at the CCCM is shown in Table 1.

**Table 1:** Therapy decisions in tumor boards and inclusion in clinical trials regarding oncological situations

	Primary	(Neo)adjuvant	Recurrence/progress	Palliative	Total
Total number of patients with therapy decision	523	444	246	314	1427 (100%)
Already included in trial	8	7	12	11	38 (2.5%)
Inclusion in clinical trial recommended by MDT	23	32	16	36	107 (7%)
Actually included	6	18	8	9	41 (2.7%)
Total patients in trial	14 (2.7%)	25 (5.6%)	20 (8.1%)	20 (6.3%)	79 (5.2%)

MTD, multidisciplinary tumor board

### Tumor board-specific evaluation

Based on the individually different organization and frequency of the respective MDT meetings at the two university hospitals, the number of presented patients differed significantly between the different MDTs in the observation. Most therapy decisions were made in the Genitourinary and Neurologic MDTs (333 and 303). In the other MDT, the number of presented cases varied between 90 and 196. Primary cases occurred most frequently in the Hematologic (49%) and Genitourinary MDTs (48%); in the other MDTs, they only accounted for about a quarter to a third of all cases (24–37%). In the Genitourinary MDT, multiple registrations were observed, mainly due to outstanding histological results. After deduction of these, the censoring rate here was 6%. In all other boards, the censorship rates were between 13 and 20%.

**Fig. 3:**
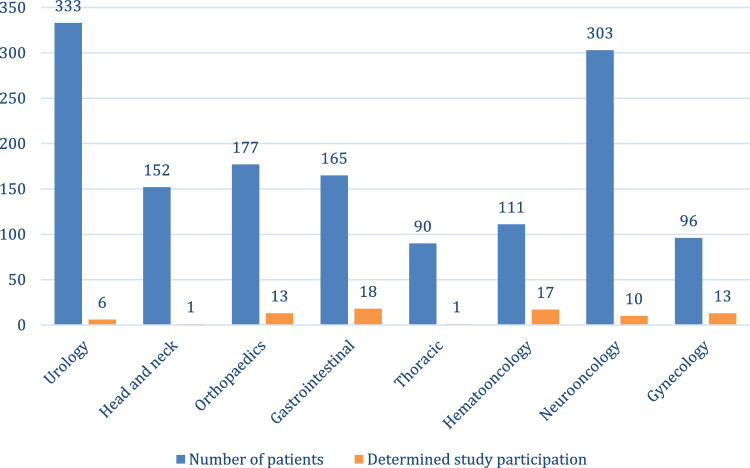
Number of patients with oncologic treatment decisions in the respective tumor boards considered and number of documented study participations

In terms of all cases considered with a treatment decision, definitive trial participation was most frequently observed in the Hematologic (15%), Gastrointestinal (11%) and Sarcoma MDT (7%). Only one trial participant each was recorded in the Thoracic and Head & Neck MDT during the observation period (Fig. 3). A summary of the oncological situations and the corresponding trial inclusions of the individual MDTs can be found in Table 2.

**Table 2:** Oncological situations and corresponding inclusion in clinical trial of the individual MDTs

MTD	Total patients	Censored	Primary	(Neo) Adjuvant	Recurrence/progress	Palliative	Final therapy in study
Genitourinary	353	20	159	116	35	23	6
Head & Neck	187	35	53	59	24	16	1
Sarcoma	216	39	44	64	30	39	13
Gastrointestinal	189	24	40	48	25	52	18
Thoracic	113	23	23	15	8	44	1
Hematologic	136	25	54	8	31	18	17
Neurologic	376	73	78	85	73	67	10
Gynecologic and Breast	227	31	72	49	20	55	13
Total	1797	270	523	444	246	314	79

MTD,  multidisciplinary tumor board.

## Discussion

With a total of 1797 case presentations, a high number of oncological cases discussed were documented in six MDT meetings, each at eight different MDTs at both university hospitals in Munich. Therefore, this can be considered representative of the annual average. Study participation was recorded immediately or in the follow-up interval for 79 (5.2%) of 1527 case discussions with treatment decisions. Internationally, the relative proportion of oncological patients treated within a therapy trial framework is also in the order of 5% (Tejeda et al. 1996; Murthy et al. 2004). For a further interpretation of these data, however, it must be considered that the trial inclusions determined via the MDTs cannot be equated with the actual relative and absolute trial participation of oncology patients at the center. The main reason for this is a high rate of trial inclusion following a case discussion in the MDT, which does not necessarily have to be based on a consensus decision in the board. The problem in determining the totality of all patients participating in trials at the entire center lies in the fact that there is no mandatory documentation of trial participation in a central trial management software. The actual rate of oncology trial participants at the observed center is therefore likely to be significantly higher than the 5.2% determined in this study.

### Challenge: decentralized trial and tumor board organization

The initiation of therapy trials or the allocation for participation in multicenter oncological therapy trials is usually carried out decentrally at large oncological centers in the study outpatient departments of individual clinics located in the center. These act as primary caregivers of the different entities and provide responsible study organizers (study doctors and nurses). Not all conference participants can be fully aware of the multitude of existing therapy trials, including numerous specific inclusion and exclusion criteria (Specchia et al. 2020). Consequently, the extent to which the existence of a potential trial is perceived and discussed for the individual patients evaluated at the MDT depends on both internal hospital communication and interdisciplinary communication. The presenter of the individual case in the MDT and the presence of the corresponding trial physicians in the respective MDT meeting play a decisive role in the discussion of possible trial participation.

The various MDTs are also mostly organized independently of each other at the oncology center and the respective leadership lies with different MDT officers acting at the centers. A possible loss of information regarding a potential trial offer is given before, during and after the respective MDT meeting. The MDT electronic data processing and administration software plays a decisive structural role in this respect (Rao et al. 2020; Hammer and Prime 2020). In most centers, this is standardized and can be viewed by all treating clinics via the hospital-wide EDP patient administration system. However, the scope of documentation both during patient registration and in the MDT itself (medical history, completeness of the clinical course, potential trial offers) is substantially based on the individual documentation culture of the individual boards (Jazieh 2011). The extent to which optional trial inclusions are queried in principle in the context of the respective case discussion is handled variably by the different MDTs. In addition, the trial inclusions made after the current case presentation in the MDT remain largely unmentioned in subsequent MDT presentations. Symptomatic of a structural loss of information is the fact that in more than 50% of the cases the reasons for a final non-inclusion in a trial could not be determined despite a MDT recommendation. In addition, the large number of patients ultimately not included in therapy trials suggests that the study design of large therapy trials is often too specific and therefore do not reflect the real situations in everyday oncology.

The independent, decentralized as well as complex study and MDT organization, thus, harbor a large pool for an information loss regarding potential trial offers in the respective case discussed in the MDT.

### Potential of tumor boards for increasing patient recruitment into oncology therapy trials

In principle, MDTs remain a valuable instrument for increasing patient recruitment into oncological therapy trials as an interdisciplinary and quality-assuring interface. Experience shows that every oncological case passes through them. Due to the above-mentioned morphological problems, this potential is currently not being fully exploited. To create continuity, transparency and quality assurance about the administration and networking of trials and MDTs, the following essential structural optimization approaches result:

- Establishment of a uniform and central trial management software for all clinics at the participating center with mandatory updating of documentation of all ongoing oncology trials and the data of included patients
- Networking of the trial management software with the hospital-wide EDP system and thus of the individual patient administration
- Uniform use of a central MDT management software for all MDTs with systematic implementation of the query of options for inclusion in therapy trials and EDP-controlled documentation of potential trials for the individual patient cases discussed with rapid access to all available trial information
- Cross-center networking of trial programs via corresponding EDP solutions and steering committees
- Reliable implementation of trial inclusion after MDT recommendation via standardized and comprehensible patient management based on "Standard Operating Procedures" (SOPs)
- The study design of future clinical trials should be adapted closer to the actual, not idealized circumstances of oncological patients. This would facilitate inclusion via MDTs.

Institutions operating across hospitals, such as CCC or general trial centers, can help researchers and clinicians to develop new therapy trials and establish the necessary contacts for their implementation by presenting the existing conditions at the responsible care structure, among other things, with management systems for clinical trials. With the establishment of uniform trial software and networking in the hospital-wide patient and MDT administration software, the prerequisite for a comprehensive and reliable flow of information about trial availability in the MDT can also be guaranteed.

In addition to these structural measures, it is vital to expand the available trial offer to increase oncological patients' recruitment rate in therapy trials. Comprehensive MDT evaluations can identify frequently represented entities and oncological situations for which the expansion of trial offers could be beneficial in particular. In the future, however, large university oncology centers will be faced with the challenge of generating extensive phase III trials to establish improved therapy standards for frequent tumor entities and large patient collectives. At the same time, the rate of highly specific, predominantly externally assigned cases is increasing (rare entities, large number of previous therapies), for which recruitment into a corresponding phase III trial is not possible. Finally, individualized medicine is steadily coming to the fore due to increasingly identified molecular-specific tumor characteristics (Molecular MDT, nNGM program) and the associated increase in tumor-specific targets (Velden et al. 2017). The increase in individually configured therapy consequently stands in the way of generating evidence-based treatment optimization for large entity-based patient collectives. An example for possible solution lies in increasing the initiation of and participation in so-called basket trials, in which the efficacy of drugs is tested for specific mutations that occur in different tumor entities.

### Limitations

There was no detailed evaluation of which entities were presented in which frequency in the individual tumor boards. In addition, it was not determined for which entities and situations therapy trials were offered at the different centers. Due to the fact that different oncology centers often treat specific entities more frequently and others less frequently than an epidemiological average, there may be distortions in the interpretation and transformation of the data to general conclusions.

## Conclusion

The potential of MDTs as an instrument for the inclusion of patients in therapy trials is high. To increase the enrollment of patients in oncological therapy trials, structural measures such as the central use of trial administration and MTB software as well as standardized tumor board discussions, must be established to ensure a seamless flow of information about actual recruiting trials and the current status of trial participation of patients.

## Data Availability

All data generated or analyzed during this study are included in this published article.
